# Is Vaccination Any Use?

**Published:** 1923-08

**Authors:** 


### Is Vaccination Any Use ?

SIR,—Is it possible for the man in the street who takes an interest in matters affecting public health to obtain any really authoritative information as to the value of vaccination ? There was a statement recently in a prominent journal that the number of deaths taking place in one year from vaccination exceed the number of deaths resulting from small-pox itself. Now, is such a statement true or not ? There are many people about to-day who have a vague idea that vaccination has been in some way discredited. This is especially so among the intellectuals who are readier to believe Mr. Bernard Shaw than Mr. Neville Chamberlain. R. E.

